# Resection of Low-Grade Gliomas in the Face Area of the Primary Motor Cortex and Neurological Outcome

**DOI:** 10.3390/cancers15030781

**Published:** 2023-01-27

**Authors:** Katharina Lutz, Levin Häni, Cédric Kissling, Andreas Raabe, Philippe Schucht, Kathleen Seidel

**Affiliations:** Department of Neurosurgery, Inselspital, Bern University Hospital, 3010 Bern, Switzerland

**Keywords:** low-grade glioma, glioma surgery, face motor cortex, intraoperative neurophysiological monitoring, GTR, motor evoked potentials, electrical stimulation

## Abstract

**Simple Summary:**

There is growing evidence that the extent of resection of low-grade glioma is directly correlated with patients’ outcomes. Preservation of function must be the other goal in brain tumor surgery. Only little is known about the organization of the face in the primary motor area (M1). New findings emphasize different motor projections to the facial motor nuclei. The aim of this retrospective study was to analyze the impact of tumor resection within the M1 face area on transient and permanent neurological deficits. Based on 12 patients, we were able to demonstrate that tumor resection within the non-dominant face motor cortex might be safe and, even in the dominant hemisphere, is only associated with transient impairment. We believe that this retrospective analysis can help identify eloquent brain areas and can lead to a change in the treatment paradigm for this disease, affecting many patients and informing many physicians worldwide.

**Abstract:**

Objective: During surgery on low-grade gliomas (LGG), reliable data relevant to the primary motor cortex (M1) for the face area are lacking. We analyzed the impact of tumor removal within the M1 face area on neurological deficits. Methods: We included LGG patients with resection within the M1 face area between May 2012 and November 2019. The primary endpoint was postoperative facial motor function. Secondary endpoints were postoperative aphasia, dysarthria, and dysphagia. Surgery was performed either with the awake protocol or under anesthesia with continuous dynamic mapping. The alarm criteria were speech arrest or a mapping threshold of 3 mA or less. Resection was completed in five patients. The resection was stopped due to the alarm criteria in three patients and for other reasons (vascular supply, patient performance) in four patients. A total of 66.7% (*n* = 8) presented with new-onset facial paresis (62.5% left LGG) and 41.7% (*n* = 5) with aphasia (all left LGG) postoperatively. After one year, all eight patients had recovered from the facial paresis. Tumor removal within the M1 face area was not associated with permanent facial motor deficits.

## 1. Introduction

Growing evidence that the extent of resection correlates with overall and progression-free survival has led to a shift toward more active treatment strategies for low-grade glioma (LGG) [1,2].

Different adjuvant therapy regimens for LGGs are debated, depending on histology, the extent of resection, and other risk factors [3,4,5]. Early adjuvant radiotherapy after surgery has demonstrated the prolongation of progression-free survival but not overall survival [4,5]. Other studies show longer progression-free and overall survival after combined radio- and chemotherapy following subtotal resection compared to radiotherapy alone [3].

Radiotherapy is a mainstay of treatment in neuro-oncology and may also affect neurological recovery. It is a challenge to achieve a high dose of the radiation field in the target volume while sparing the organs at risk [6]. Pintea et al. showed better motor outcomes for tumors in the primary motor cortex (M1) after surgical resection than after radiotherapy [7]. This may indicate that M1 should be treated as an organ at risk. Therefore, the aim should be to achieve the greatest possible tumor reduction in M1 to minimize the postoperative radiation dose.

To balance radical resection against preservation of function, information about eloquence is essential. Various tools aim to identify functionally eloquent areas to maximize the extent of resection while minimizing the risk of a neurological deficit postoperatively [8]. Most studies concerning motor eloquence have focused on deficits in the extremities. However, little is known about the motor organization of the face in M1. Data suggest that the connection pathways may differ from those of other M1 areas [9].

After infarction of the medial cerebral artery (MCA), patients experienced contralateral impairment of facial muscle movements, especially in the lower face [10]. The sparing of the upper face has been explained by bilateral cortical innervation to the corresponding part of the facial nuclei. New findings emphasize different motor projections to the facial motor nuclei [11,12]. However, data on tumor surgery in the primary motor cortex for the face area are sparse, likely due to the low incidence of diagnosed and surgical treated LGG which only extend in the M1 face area. Thus, reported series are restricted to case reports [13,14]. These have suggested the maintenance of mouth motor function after resection of the non-dominant face motor area [15,16,17].

The objective of our study was to analyze the impact of tumor resection within the M1 face area on transient and permanent neurological deficits.

## 2. Methods

### 2.1. Study Design

We retrospectively screened all patients who had undergone resection of an LGG (WHO grade 2) in the frontal lobe between May 2012 and November 2019 at our institution. We included patients with: (i) a diagnosis of LGG (astrocytoma and oligodendroglioma, WHO grade 2, all isocitrate dehydrogenase (IDH) mutated); (ii) existing pre- and postoperative magnetic resonance imaging (MRI); (iii) involvement of the M1 face area; and (iv) resection of the glioma within the face motor area. Patients with a non-contrast-enhancing tumor on MRI, which was defined based on the final histopathological analysis as an anaplastic glioma (WHO grade 3) were excluded because of the possible more aggressive behavior of WHO grade 3 tumors. It would be difficult to reliably distinguish between deterioration or stagnation of neurological outcome due to surgical sequelae and tumor progression.

Approval was obtained from the local ethics committee.

The primary endpoint of our study was facial motor function. Secondary endpoints were the occurrence of aphasia, dysarthria, and dysphagia. Aphasia was defined as an impairment in speech production or comprehension. It was divided into three categories: 1. “mild aphasia”, with difficulty finding the right words; 2. “moderate aphasia”, with non-meaningful sentences or low word production; and 3. “severe aphasia”, with no or only unrecognizable word production. Dysarthria was considered a disorder of articulation related to a weakness of the articulation muscles. Retrospective data were evaluated, i.e., this information was taken from patient records.

We also analyzed the influence of hemispheric dominance, age, sex, histopathology, recurrence, epileptic seizures (pre-, intra-, and postoperative), tumor localization, and type of surgery (awake surgery versus general anesthesia with motor mapping) on primary and secondary outcomes. The hemispheric dominance was determined on the basis of anamnestic data, neuropsychological reports, and fMRI data.

### 2.2. Surgical Procedure and Intraoperative Neurophysiological Monitoring

We applied our standard protocol for intraoperative neurophysiological monitoring and mapping to all patients. This included direct cortical stimulated monitoring (DCS) of motor evoked potentials (MEP) via a strip electrode on the precentral gyrus (DCS MEP), cortical short-train low-threshold mapping with a monopolar probe, and continuous dynamic subcortical short-train mapping via the suction probe; details are published elsewhere [18,19,20]. We recorded MEPs from at least three to four facial muscles, including orbicularis oculi and oris, masseter, and tongue. In patients who underwent awake surgery, we applied Penfield stimulation with simultaneous electrocorticogram (ECoG) recordings [21].

The neurophysiological alarm criteria for the awake surgery setting were either speech arrest or dysarthria. For the protocol under total intravenous anesthesia, it was either a significant alteration in DCS MEP monitoring or a subcortical monopolar cathodal short-train mapping threshold of 3 mA or below during subcortical dynamic mapping [18,20,22].

Patients who had tumors near presumed speech areas in the dominant hemisphere underwent awake surgery, if feasible, to allow for speech mapping. A two-step model was used for the tumor resection. In the first step, the patients were awake for the speech mapping. In the second step, the patients were intubated and the mapping for the motor function was carried out while they were asleep.

All other patients underwent surgery under general anesthesia with continuous dynamic mapping. Glioma resection was performed according to our standards for microsurgical techniques, and neurophysiological and image guidance [18,23]. In almost half of those patients, we performed a navigated transcranial magnetic stimulation (TMS) examination to locate the face motor cortex [24].

### 2.3. Postoperative Outcome and Clinical Evaluation

A board-certified physician (the attending neurosurgeon) evaluated the postoperative neurological deficit one day, one week, three months, and one year after surgery, as is standard practice at our institution. The primary outcome was the face motor function measured by the House–Brackmann six-point scale. Grade 1 indicates normal function, whereas grade 6 indicates total paralysis [25]. Grade 1 was considered to indicate complete recovery.

### 2.4. Statistical Analysis

For the collection of data on facial paresis and aphasia, an ordinal scale was applied. Dysarthria and dysphagia were assessed as binary variables. We divided aphasia into three categories: no aphasia, mild aphasia, and severe aphasia. SPSS Version 25 (Statistical Package for the Social Sciences) was used for statistical analysis. Between-group comparisons were made by using a chi-square test or Fisher’s exact test, as appropriate. A *p*-value < 0.05 was considered significant.

## 3. Results

### 3.1. Patient Population

Between May 2012 and November 2019, a total of 105 patients with either an astrocytoma or an oligodendroglioma grade 2 in the frontal lobe underwent tumor resection. Of these, 12 patients (11.4%) presented with a LGG involving the M1 face area and were included in the study. All of them underwent resection with excision of the tumor within the M1 face area, as confirmed by postoperative MRI. An astrocytoma grade 2 was confirmed in 6 patients and an oligodendroglioma grade 2 in the other six. Half of the patients included were undergoing reoperation due to tumor recurrence.

The mean age of the patients was 38 years (range: 19–63 years), and 41.7% were female. In 5 patients (41.7%), the glioma was located on the left side. A total of 7 patients underwent surgery for a glioma on the right side (58.3%). Of the 12 patients (8.3%), 1 was left-handed and had a tumor located in the right non-dominant hemisphere. All other patients were right-handed, and all of them were considered non-dominant. A total of 11 patients (91.7%) were diagnosed following new-onset seizures. One patient had received an MRI for headaches. Resection was complete in 5 patients (see Supplementary Material Table S1).

### 3.2. Intraoperative Findings

A total of 3 patients (25%) had undergone surgery while awake or in a combined procedure of an initial awake stage for speech testing, followed by an asleep stage with mapping for motor function. In all three cases, resection was incomplete due to positive stimulation of cortical speech sites or the subcortical arcuate fascicle. Because of the expected poor compliance (see below), the other two patients with a tumor on the left side were not considered eligible for an awake procedure.

**Figure 1:**
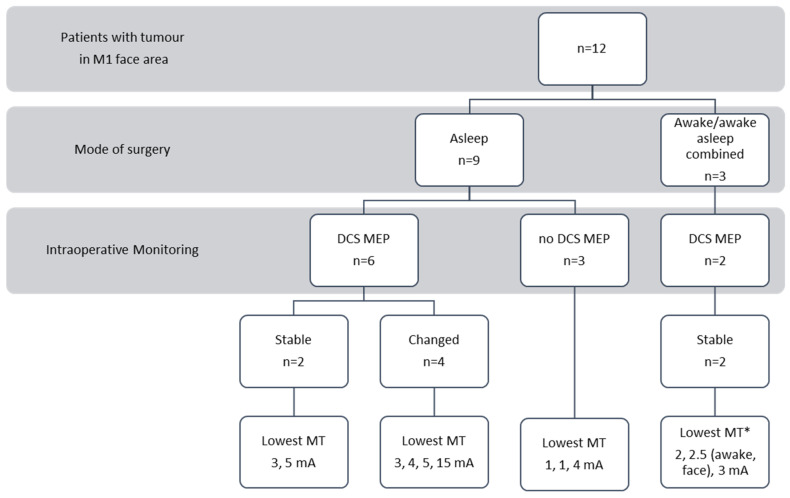
Intraoperative neurophysiological settings and paradigms: patient distribution intraoperatively for awake or general anesthesia. 1. For awake surgery, Penfield stimulation (50 Hz, bipolar probe) was used. 2. For asleep surgery, short-train monopolar stimulation was used (see Methods for details). DCS—direct cortical stimulation; MEP monitoring—motor evoked potential; MT—motor threshold (lowest stimulation intensity that elicits a subcortical MEP); mA—milliampere; and * 2 patients mapped asleep and one awake for face stimulation.

A total of 9 patients (75%, 2 with a left-sided tumor and 7 with a right-sided tumor) underwent surgery with continuous subcortical mapping under general anesthesia. The lowest subcortical mapping thresholds are presented in Figure 1 and Table 1. In five patients, the resection was stopped intraoperatively because it was judged complete (Table 1). In two patients, the resection was halted because of the vascular perforators of the MCA at the level of the basal ganglia. Furthermore, in two patients, the resection was aborted due to the presumed proximity of cortical eloquent speech areas or the arcuate fascicle. These 2 patients were operated on under general anesthesia either because of preoperative aphasia (patient number 7) or patient non-compliance in the awake craniotomy setting (patient number 12). These two patients were the oldest in the series (Table 1).

**Figure 2:**
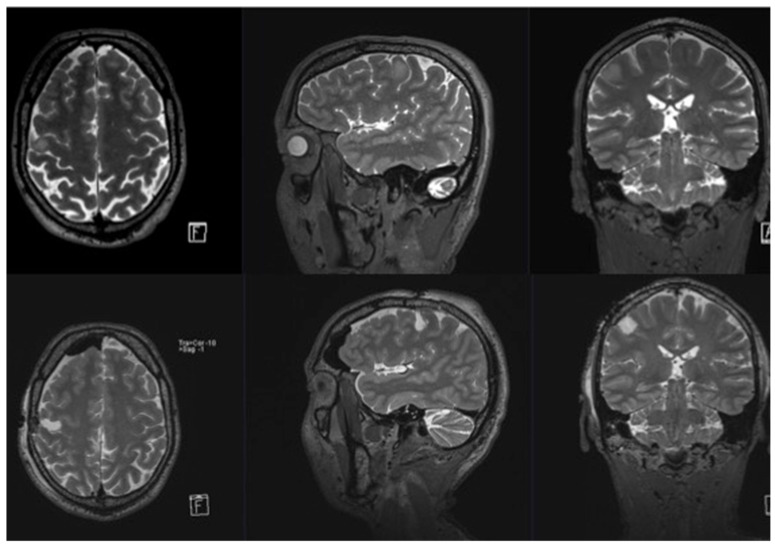
Pre- and postoperative magnetic resonance imaging (MRI) of patient number 8. **Upper row**: preoperative axial, sagittal, and coronal T2 MRI images illustrating the tumor location in the medial frontal gyrus and its infiltration of the face motor cortex. The tumor was resected within the face motor area with intraoperative neurophysiological MEP monitoring, direct cortical stimulation, and continuous dynamic mapping with a low subcortical mapping threshold of 4 mA. **Lower row**: postoperative axial, sagittal, and coronal T2 MRI images illustrating the tumor resection cavity. For more clinical details, see Table 1 and Table 2.

**Figure 3:**
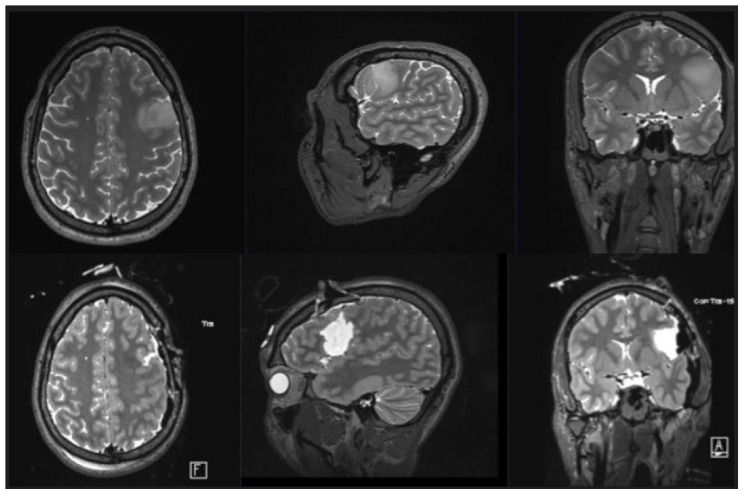
Pre- and postoperative magnetic resonance imaging (MRI) of patient number 4. **Upper row**: preoperative T2 axial and sagittal MRI images illustrating the tumor location in the medial and inferior frontal gyrus. The patient underwent awake surgery. After mapping the cortex and visualization of several speech areas, a corticotomy and subpial resection of the tumor were performed. The tumor was removed, reaching a subcortical stimulation threshold of 2 mA for the facial muscles. **Lower row**: postoperative T2 axial and sagittal MRI slices illustrating the tumor resection cavity in the medial and inferior frontal gyrus on the right hemisphere with resection in the primary motor cortex of the face area.

Penfield stimulation (50 Hz, bipolar probe) was used during awake surgery. Short-train monopolar stimulation was used during surgery under general anesthesia (see Methods for details). mA—milliampere. Two illustrative cases are presented in Figure 2 and Figure 3.

### 3.3. Clinical Findings

#### 3.3.1. Facial Paresis

None of the patients had facial nerve palsy before surgery. After surgery, 66.7% of the patients (*n* = 8) presented with new-onset facial paresis (Table 1 and Table 2). All patients who underwent surgery on the left (dominant) hemisphere (*n* = 5, 100% of left-sided tumors) showed facial paresis, compared to 3 of 7 patients with a right (non-dominant) tumor location (43% of the right-sided tumors) (Table 2). Localization of the tumor in the left (dominant) hemisphere was associated with a trend towards a higher likelihood of a transient postoperative deficit of the face muscles (*p* = 0.081). After 3 months, only 2 patients (25%) still showed residual paresis (1 with the tumor located on the right side, 1 on the left). After 1 year, all 8 patients (100%) had recovered from the paresis [26].

In 3 patients, additional involvement of the premotor face area was seen (patient number 1, 7, 12). Regarding postoperative facial motor outcome, we could not find a significant difference.

Four of the six oligodendrogliomas were located in the left hemisphere, and all patients with these left-sided gliomas developed a new facial deficit. Histology did not seem to have any influence on the occurrence of new postoperative facial deficits (*p* = 1.00, Table 2).

Division of the patients into 2 groups based on the mean age (38 years) found no significant effects of age. Younger patients (<38 years) more often developed a facial deficit compared to older (>38 years) patients (83.3% versus 50%, *p* = 0.545). However, younger patients recovered faster than older patients: 60% versus 0% had recovered from their deficit after 1 week, and 100% versus 33.3% after 3 months.

#### 3.3.2. Aphasia

Before surgery, there was only one patient with a left-sided tumor who had aphasia (patient number 7). Postoperatively, 41.7% of patients (*n* = 5) presented with aphasia (for information on patients with severe aphasia, see Table 2), of whom 80% showed improvement after 1 year. All patients with a speech deficit after surgery had a glioma in the dominant hemisphere (*p* = 0.001). After 1 year, 1 patient still had severe aphasia (patient number 7). This was the only patient who already had mild aphasia before surgery. One other patient who did not have aphasia before surgery had severe aphasia postoperatively. However, after 1 year, his aphasia was only mild, and his recovery was classified as good (patient number 12).

Hemispheric dominance was the only risk factor for postoperative aphasia (Table 1 and Table 2). Patients with an oligodendroglioma seemed to be at higher risk for postoperative aphasia (66.7% versus 16.7% in patients with astrocytomas, *p* = 0.242). This could reflect exclusively the distribution of the location in the dominant hemisphere, since four out of six oligodendrogliomas were located in the dominant hemisphere, whereas this was true for only one out of six astrocytomas. Therefore, histopathology seemed to be a dependent variable that was confounded by the distribution of tumor location.

#### 3.3.3. Dysarthria

No patient presented with dysarthria preoperatively. After glioma surgery, 16.7% of patients (*n* = 2, one in each age group) had dysarthria (Table 1 and Table 2). Both patients recovered fully, 1 (patient number 3, age 33 years) recovered within 1 week and the other (patient number 12, age 53 years) within 3 months.

#### 3.3.4. Dysphagia

Before surgery, no patient suffered from dysphagia. One patient (8.3%, patient number 4, age 24 years) had dysphagia after surgery (Table 1 and Table 2) and recovered fully within 1 week.

## 4. Discussion

The neurooncological challenge is to understand presumed eloquence in the precentral gyrus, especially in the face motor area.

### 4.1. Why Do All Patients Recover from Facial Palsy?

In our surgical series of 12 patients who underwent resection surgery on LGG within the M1 motor face area, 100% had full facial motor function (House–Brackmann I) at 12 months. A total of 8 of the 12 patients (66.7%) had facial palsy immediately after surgery. In 3 of these patients (37.5%), the palsy might have been due to the surgical manipulation of tissue, as they recovered completely before discharge. Of the remaining 5 patients (62.5%), 3 recovered within 3 months, and the other 2 within 1 year.

A possible bilateral innervation of the facial nucleus might explain the good neurological long-term outcome [27,28]. However, this mechanism alone does not explain (a) why patients still experienced a transient deficit and (b) why they were more affected when the dominant M1 face area was resected.

It is generally accepted that the supranuclear innervation of the seventh nerve is different for the upper and lower face muscles [29]. The bilateral innervation of the muscles of the forehead is well known [10,27,29]. The innervation of the lower face is assumed to be only from the contralateral hemisphere [28]. Nevertheless, there are data suggesting that some neurons of the facial nucleus that are responsible for the lower face have projections from other areas. A direct cortico-cortical connection between the supplementary motor area (SMA) and M1 as the main influence on motor output (corticospinal tract, CST) has also been described [30,31,32]. Other areas, such as the ventral lateral premotor cortex and the SMA, are involved in voluntary facial motor expression [29]. Facial expression is not only voluntary, as “reflexive” patterns exist that are controlled by areas in the brainstem [29].

Information on the neurological outcome after resection of the M1 face area is scarce. Leroux et al. reported similar results in two patients with a non-dominant glioma who had undergone a total resection in the face motor cortex. They had postoperative transient facial weakness and transient facial apraxia [16]. The authors concluded that a resection in the face motor area on the non-dominant side is safe due to the bilateral representation of face motor function at the neocortical level. In contrast to our findings, they speculated that the dominant motor face areas should not be resected because of language-related structures and motor apraxia. However, they did not present a case supporting their speculation or discuss whether this motor apraxia might be transient or permanent.

Fontaine et al. reported similar results in 11 patients after resection of the SMA. No language difficulties were noted after resection of the right, non-dominant, SMA for the M1 face area [33]. The sparing of the motor cortex was confirmed at the end of surgery by direct electrical stimulation. Interestingly, they saw contralateral facial deficits only after resection of the left SMA. No patient with a right-sided tumor showed a facial motor deficit after surgery, despite the occurrence of transient limb paresis [33]. These findings may elucidate the difference in the organization of the face motor control in the SMA compared to the motor control of the extremities. However, this study only addressed SMA lesions and did not describe the involvement of the M1 face area of the precentral gyrus.

### 4.2. Bilateral Innervation Versus Plasticity

#### 4.2.1. Bilateral Innervation

As already mentioned, Leroux et al. suggested that a transient deficit could be due to a bilateral innervation and hence compensation by the M1 of the healthy hemisphere [16]. This does not explain why there would be any deficit after contralateral resection with an intact ipsilateral pathway. The patients should wake up from surgery neurologically intact. The existence of bilateral innervation with one “main path” and some kind of reactivation of a different “secondary” path after resection or damage to the main innervation could be considered [34]. Innervation from outside M1 (supplementary motor and rostral cingulate motor cortex) and a (re-)activation of these pathways could explain the ability to express emotion through facial movements after a middle cerebral artery stroke [27].

There is evidence to support such a (re-)activation: a higher activation in the contralateral hemisphere in M1 and bilateral premotor areas after stroke was found in a meta-analysis [35]. However, this mechanism alone does not predict a good outcome and motor recovery seems far more complex [35].

Earlier TMS studies showed a bilateral response of the lower facial muscles after TMS application to the motor cortices [36]. There were 2 phases of motor responses with different latencies (short latency of 7.6 ms and longer latency > 15 ms). The authors interpreted the faster response as the activity in a predominantly contralateral oligosynaptic corticobulbar pathway and the slower response as a polysynaptic indirect (e.g., cortico-tegmento-nuclear) bilateral pathway [36]. This might be the result of volume conduction and activation of the contralateral facial muscles’ bilateral innervation [37], especially because measurable activity of the facial muscles on both sides was recorded during rest. Yildiz and colleagues tried to minimize volume conduction with their electrode placement and by optimizing their protocol, with similar results. Another explanation might be spreading to the supraorbital nerve with consecutive activation of the blink reflex, which could only explain the responses of the upper facial muscles. However, after central facial paresis, patients showed a decreased EMG amplitude on the side of the paresis after TMS application for both hemispheres [37].

#### 4.2.2. Imbalance in Inhibition and Excitation

Another explanation is based on the inhibition and excitation of different brain regions after an injury to M1 (due to a stroke or the removal of a tumor). Hallet et al. [38] proposed that the interaction of computational modules located in adjacent and remote interconnected brain regions was involved in impairment and recovery after stroke [38].

Finally, there might be a shift from contralateral activation in the healthy motor system to a more bilateral activation pattern [39,40]. However, activation of the contralesional (non-affected) M1 has been negatively correlated with motor performance (maladaptive mechanism) in other studies [38]. This may even result in an increase of inhibitory influence from the contralesional M1 on the ipsilesional M1, which negatively affects motor recovery [38]. Alternatively, associative motor areas, such as the ipsi- or contralesional frontal dorsal premotor area, might play a supportive role in promoting motor recovery [38,41]. These network interactions may change over time, which could explain the dynamic aspect of recovery [38].

#### 4.2.3. Plasticity

Recovery might reflect a change in neuronal function in other brain areas to cover the function of the resected cortex—a representational remodeling [42], also known as brain plasticity. Plasticity as the reason for the good outcome would help explain the occurrence of deficits postoperatively, and the time to recovery might be interpreted as a “training period”.

However, more than 50% of patients recovered from facial motor weakness in less than 1 week. This contradiction between the theory that new connections grow in brain areas after damage to function and the theory of regaining old pathways was described in 1993 for the somatosensory cortex [43]. The somewhat short period until full recovery may be explained by short-, middle-, and long-term remodeling [44]. SMA-syndrome recovery is already known to take place within a few weeks [44]. Duffau explains good recovery after resection of motor areas in terms of different stages of plasticity [44]. In summary, it differentiates between pre-, intra-, and postoperative brain plasticity with a dynamic reorganization of eloquent maps induced by slow-growing tumors such as low-grade gliomas [44].

Duffau’s theory is that recovery occurs in accordance with a hierarchically organized model. In the preoperative state, he describes three possible scenarios: first, if the function persists within the tumor, after resection there will be a new (persistent) deficit. Second, if the function has already been redistributed around the tumor preoperatively, a transient deficit will occur, with recovery occurring within a few weeks to months. Third, there could be remote areas within the ipsilateral hemisphere or even the contralateral hemisphere, which might take over the function so that after the removal of the tumor located in the eloquent brain area only a mild or short-term transient deficit will be seen [44].

Intraoperatively, Duffau distinguishes between loco-regional hyper-excitability and acute unmasking of redundant areas (pre- and post-Rolandic functional bilocation) [44]. For the postoperative state, he outlined the participation of the contralateral hemisphere as a post-surgical functional compensation, so, with the reactivation of latent networks, there is no definite deficit [44]. Postoperative swelling caused by surgical manipulation could also account for the rather rapid recovery within 1 week.

#### 4.2.4. Patient Age and the Likelihood of Recovery—Is There Any Connection?

A possible trend towards faster recovery in the younger patients of our series could favor plasticity as a key mechanism of recovery. We also found that younger patients more often had a postoperative deficit. This could be due to a more radical surgical strategy to maximize the extent of resection in this group with a higher life expectancy. Old age could be a risk factor for slower recovery from all deficits. A worse clinical outcome in older patients with LGG has previously been reported [45]. Poorer recovery after stroke might reflect a reduced capacity for plasticity in the elderly [46]. EEG studies have verified a progressive disconnection and therefore a decrease in plasticity in older patients [38].

All our patients had a low-grade tumor, and owing to the slow growth, the brain would already have had time for reorganization and building new pathways for function before surgery [47]. Therefore, the reason for good recovery, whether due to bilateral innervation, plasticity, or both, is still not completely explained. More studies with prospective data are needed, including pre- and postoperative fMRI or navigated TMS to investigate the excitability of the ipsi- and contralateral M1 face areas.

### 4.3. The Role of Dominant Versus Non-Dominant Tumour Location

The role of the dominant hemisphere in relation to recovery from facial deficits is not clear. Resection of the face motor area on the dominant hemisphere seems to carry a higher risk for aphasia and dysarthria postoperatively. Nevertheless, in our small series, only 2 patients presented with language difficulties lasting more than 3 months after surgery, and 1 of them had only slight fluency problems after 1 year.

We were able to show that although the dominant hemisphere might be a risk factor for transient neurological deficits full recovery is achieved after one year in all patients.

### 4.4. Limitations of the Study

We presented a small series of 12 patients. The retrospective study design might have led to selection and observer bias. The small sample size might be attributed to the fact that the incidence of diagnosed and surgical treated LGG in the face motor area is low. It is evident that larger prospective series are needed to draw a definitive conclusion. However, to our knowledge, this is the first homogeneous series addressing the topic of resection of LGG in the face motor area with long-term results.

Furthermore, due to the retrospective design, detailed aphasia scores were not reported. To overcome this limitation, we classified aphasia as mild, moderate, and severe. Anyhow, our primary objective of this study was to assess the impact of resection of the face M1 area on facial motor function, which was assessed in detail by the HBS.

## 5. Conclusions

**Figure 4:**
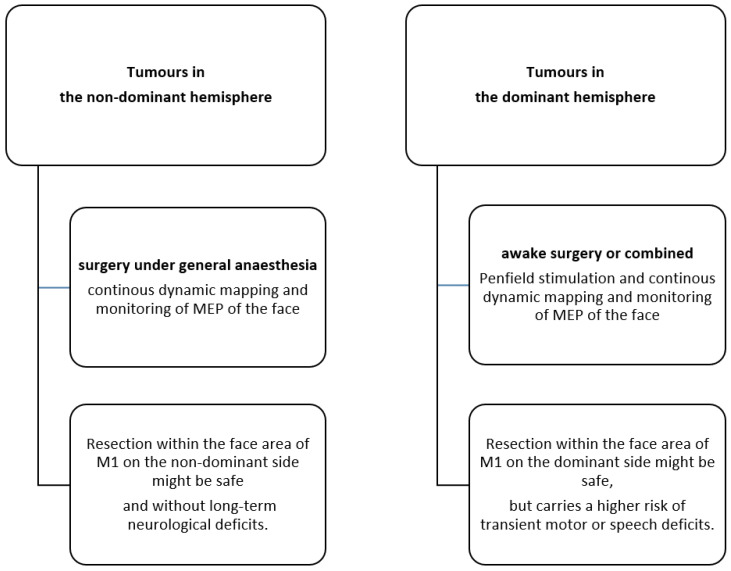
Suggested paradigm: comparison of the neurological outcome of tumors in the dominant and non-dominant hemispheres.

In our series of LGG, tumor removal within the M1 face area was not associated with permanent facial motor deficits. Hemispheric dominance was a strong predictor of the occurrence of postoperative transient deficits. Thus, resection within the non-dominant face motor cortex might be safe and, even in the dominant hemisphere, is only associated with transient impairment (Figure 4). However, larger series are needed before a definitive statement can be made.

## Figures and Tables

**Table 1:** Intraoperative electrophysiology findings and pre- and postoperative deficits (directly after surgery).

				Pre- and Postoperative Deficits				Tumor Volume	
Patient No.	Hemisphere/Dominance	Age (yrs.)	Lowest Motor Threshold (mA)	Facial Paresis	Aphasia	Dysarthria	Dysphagia	Preoperative (mL)	Postoperative (mL)
1	left (dominant)	29	2.5 (awake)	no/yes	no/yes	no/no	no/no	103.54	45.87
2	right (non-dominant)	40	1	no/no	no/no	no/no	no/no	7.29	<1
3	left (dominant)	33	5 (awake: 3)	no/yes	no/yes	yes/yes	no/no	28.23	9.53
4	left (dominant)	24	2 (awake: 2)	no/yes	no/yes	no/no	no/yes	37.78	1.52
5	right (non-dominant)	22	1	no/no	no/no	no/no	no/no	17.27	<1
6	right (non-dominant)	34	5	no/yes	no	no/no	no/no	28.43	7.16
7	left (dominant)	63	15	no/yes	yes/yes	no/no	no/no	9.13	2.11
8	right (non-dominant)	48	4	no/no	no/no	no/no	no/no	1.14	<1
9	right (non-dominant)	41	3	no/yes	no/no	no/no	no/no	2.25	<1
10	right (non-dominant)	51	4	no/no	no/no	no/no	no/no	24.36	3.14
11	right (non-dominant)	19	3	no/yes	no/no	no/no	no/no	2.92	<1
12	left (dominant)	53	5	no/yes	no/yes	no/yes	no/no	33.81	13.73

**Table 2:** Number of patients with neurological deficits over time in relation to dominance, histology, and age (*n* = 12).

	Facial Weakness Postoperative				Aphasia (Severe) Postoperative				Dysarthria Postoperative				Dysphagia Postoperative			
	1 Day	1 Week	3 Month	1 Year	1 Day	1 Week	3 Month	1 Year	1 Day	1 Week	3 Month	1 Year	1 Day	1 Week	3 Month	1 Year
*Dominance (tumor location)*																
Non-dominant (*n* = 7)	3	2	1	0	0	0	0	0	0	0	0	0	0	0	0	0
Dominant (*n* = 5)	5	3	1	0	4	3	2	1	2	1	0	0	1	0	0	0
*Histology*																
Astrocytoma WHO ° 2 (*n* = 6)	4	2	1	0	1	1	0	0	0	0	0	0	1	0	0	0
Oligodendroglioma WHO ° 2 (*n* = 6)	4	3	1	0	3	2	2	1	2	1	0	0	0	0	0	0
*Age*																
<38 years (*n* = 6)	5	2	0	0	2	1	0	0	1	0	0	0	1	0	0	0
>38 years (*n* = 6)	3	3	2	0	2	2	2	1	1	1	0	0	0	0	0	0

WHO—World Health Organization, °—Grade.

## Data Availability

All data relevant to this study are included in the article.

## Supplementary Materials

The following supporting information can be downloaded at: https://www.mdpi.com/article/10.3390/cancers15030781/s1, Table S1: Extent of resection and histology (*n* = 12).
