# Supplementary material for: Resection of Low-Grade Gliomas in the Face Area of the Primary Motor Cortex and Neurological Outcome
Source: Cancers (Basel). 2023 Jan 27;15(3):781. doi: 10.3390/cancers15030781 (PMC9913697; doi:10.3390/cancers15030781)
Supplement: Supplementary file 1 [file cancers-15-00781-s001.zip › cancers-2076849-supplementary.pdf]

Table S1. Extent of resection and histology (n=12)

Patient No.	histology	Extent of resection
1	oligodendroglioma WHO grade 2	STR
2	oligodendroglioma WHO grade 2	GTR
3	oligodendroglioma WHO grade 2	STR
4	astrozytoma WHO grade 2	STR
5	astrozytoma WHO grade 2	GTR
6	astrozytoma WHO grade 2	STR
7	oligodendroglioma WHO grade 2	STR
8	astrozytoma WHO grade 2	GTR
9	astrozytoma WHO grade 2	GTR
10	oligodendroglioma WHO grade 2	STR
11	astrozytoma WHO grade 2	GTR
12	oligodendroglioma WHO grade 2	STR

GTR=gross total resection
STR=subtotal resection
